# Nrf2 signaling pathway: current status and potential therapeutic targetable role in human cancers

**DOI:** 10.3389/fonc.2023.1184079

**Published:** 2023-09-22

**Authors:** Li Lin, Qing Wu, Feifei Lu, Jiaming Lei, Yanhong Zhou, Yifei Liu, Ni Zhu, You Yu, Zhifeng Ning, Tonghui She, Meichun Hu

**Affiliations:** ^1^ Key Laboratory of Environmental Related Diseases and One Health, School of Basic Medical Sciences, Xianning Medical College, Hubei University of Science and Technology, Xianning, China; ^2^ Department of Medical School of Facial Features, Xianning Medical College, Hubei University of Science and Technology, Xianning, China; ^3^ School of Biomedical Engineering, Xianning Medical College, Hubei University of Science and Technology, Xianning, China

**Keywords:** Nrf2, Nrf2 signaling pathway, oxidative stress, cancer, therapeutic target

## Abstract

Cancer is a borderless global health challenge that continues to threaten human health. Studies have found that oxidative stress (OS) is often associated with the etiology of many diseases, especially the aging process and cancer. Involved in the OS reaction as a key transcription factor, Nrf2 is a pivotal regulator of cellular redox state and detoxification. Nrf2 can prevent oxidative damage by regulating gene expression with antioxidant response elements (ARE) to promote the antioxidant response process. OS is generated with an imbalance in the redox state and promotes the accumulation of mutations and genome instability, thus associated with the establishment and development of different cancers. Nrf2 activation regulates a plethora of processes inducing cellular proliferation, differentiation and death, and is strongly associated with OS-mediated cancer. What’s more, Nrf2 activation is also involved in anti-inflammatory effects and metabolic disorders, neurodegenerative diseases, and multidrug resistance. Nrf2 is highly expressed in multiple human body parts of digestive system, respiratory system, reproductive system and nervous system. In oncology research, Nrf2 has emerged as a promising therapeutic target. Therefore, certain natural compounds and drugs can exert anti-cancer effects through the Nrf2 signaling pathway, and blocking the Nrf2 signaling pathway can reduce some types of tumor recurrence rates and increase sensitivity to chemotherapy. However, Nrf2’s dual role and controversial impact in cancer are inevitable consideration factors when treating Nrf2 as a therapeutic target. In this review, we summarized the current state of biological characteristics of Nrf2 and its dual role and development mechanism in different tumor cells, discussed Keap1/Nrf2/ARE signaling pathway and its downstream genes, elaborated the expression of related signaling pathways such as AMPK/mTOR and NF-κB. Besides, the main mechanism of Nrf2 as a cancer therapeutic target and the therapeutic strategies using Nrf2 inhibitors or activators, as well as the possible positive and negative effects of Nrf2 activation were also reviewed. It can be concluded that Nrf2 is related to OS and serves as an important factor in cancer formation and development, thus provides a basis for targeted therapy in human cancers.

## 1 Introduction

Cancer is commonly known as a kind of malignant tumors and leads to massive human death. Enormous studies have reported that over the next 2 decades, the number of new cancer cases is expected to rise approximately 50% in the whole world (1). According to global cancer data, 9.96 million people worldwide will die by 2020, of which China ranks first in terms of cancer deaths (2). Due to its high metastasis, infinite proliferation, immune escape and high mortality characteristics, current cancer treatments include surgical intervention, radiation, and taking chemotherapeutic drugs (3). Most of these classic non-operative anticancer treatments kill tumor cells by influencing cancer severely associated oxidative stress (OS) (4).

The so-called OS refers to the generation of free radicals and reaction metabolites that cause the imbalance between oxidation and antioxidant action in the body, resulting in the production of many oxidation products, such as reactive oxygen species (ROS) or reactive nitrogen species (RNS) production, causing protein oxidation, DNA destruction, etc., then make the normal tissues finally proceed to diseases (5). OS may be ubiquitous in multiple organ systems of the human body and cancer can be induced if the human body is in a state of OS for a long time. OS will lead to chronic inflammation, thereby mediating the occurrence of chronic diseases, including cancer (6). In addition, sustained OS can activate a variety of transcription factors, including NF-κB, AP-1, p53, HIF-1α, and Nrf2. Activation of these transcription factors will trigger their downstream effectors including growth factors, inflammatory cytokines, chemokines, cell cycle regulatory molecules and anti-inflammatory molecules (7). All these transcription factors and downstream effectors may arouse excessive activation of inflammatory mediators and promote tumor development (8).

As a key transcription factor for anti-oxidative stress, nuclear factor-E2-related factor 2 (Nrf2) exerts important multifunction (9). Firstly, Nrf2 is mainly responsible for protecting cells from OS. Playing a crucial role in preventing apoptosis, inflammation and tumors, it is the most important intrinsic anti-oxidative stress pathway discovered so far (10). Reports have shown that the activation of Nrf2 can prevent many chronic diseases, including cardiovascular disease, respiratory diseases and neurodegenerative diseases (11, 12), as well as cancer. Secondly, Nrf2 is also an important regulator that promotes the transition of the cell cycle from G2 phase to M phase. When Nrf2 function is damaged, cells cannot enter M phase normally, the mitosis process is forced to stop, and cell proliferation is interrupted (13). In addition, cancer cells require higher energy and anabolism to support their rapid cell growth, and Nrf2 helps meet these demands, thus promoting cancer progression (14, 15).Therefore, Nrf2 can not only act as a tumor-promoting gene, but also act as a tumor suppressor to play an anti-tumor role. The dysregulation and activation of the Nrf2 system is one of the main reasons for the pathogenesis of cancer (16).

Under the focus of targeted therapy of oncology, researches on the carcinogenesis associated with Nrf2 are increasingly to the concern (9). Targeted therapy is a new treatment developed directly for molecular abnormalities or tumor cells that lead to the development of cancer. Meanwhile, the dual functions of Nrf2 in cancer have been profoundly elucidated, and the Nrf2 signaling pathway has become an important therapeutic target for treating cancers, neurodegenerative diseases and many autoimmune or inflammatory diseases (17, 18). Here in this research, we systematically review the function and role of Nrf2 signaling pathway in several typical malignant tumors, summarize the recent progress of Nrf2 in cancer treatment and elaborate the current status and potential therapeutic targetable role of Nrf2. This research will provide a basis for Nrf2’s use as a potential therapeutic target for combating tumors in the future.

## 2 Structure and function of Nrf2

**Figure 1:**
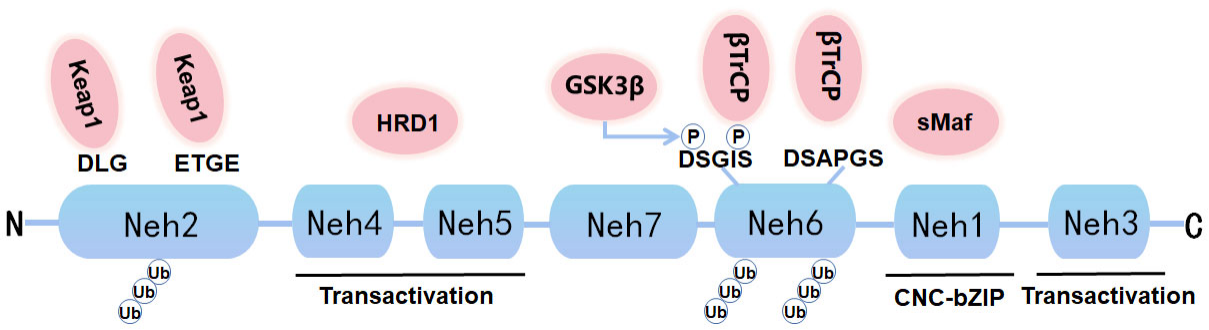
Graph for Nrf2 protein structure. Nrf2 is composed of seven Neh domains representing different functions. N-terminal Neh2 domain has DLG and ETGE motifs. The transactivation of Nrf2 is mediated by Neh4, Neh5 and Neh3. Neh6 has DSGIS and DSAPGS motifs, which can mediate Keap1 independent proteasome degradation of Nrf2. Neh1 has a bZIP motif that binds to ARE during dimerization with sMaf.

Nrf2, a soluble protein found primarily in the cytoplasm, belongs to the Cap ‘n’ Collar (CNC) subfamily, comprises in 605 amino acids and contains seven specific functional related protein homology domains (Nrf2-ECH homology, Neh) (**Figure 1**). The Neh1 with ubiquitin-conjugating enzyme to enhance the stability and transcriptional activity of Nrf2. Neh2, the second domain, contains two motifs known as DLG and ETGE, which can be identified and interacted with Kelch-like ECH-associated protein 1 (Keap1). Among them, Keap1 is a substrate adaptor for cullin-based E3 ubiquitin ligase, which inhibits the transcriptional activity of Nrf2 via ubiquitination and proteasomal degradation under normal conditions (19). Neh3, Neh4, and Neh5 act as transactivation domains. The Neh3 region is located at the-COOH end of Nrf2 and is transcriptionally activated by binding to a specific ATPase/helicase DNA-binding protein (CHD) 6; Neh4 and Neh5 are located between Neh1 and Neh7. Neh4 and Neh5 are required to activate cAMP response element binding protein to initiate the transcription process. The sixth domain-Neh6, contains a peptide region that is rich in serine amino acids, and contains two motifs known as DSGIS and DSAPGS. Besides, Neh6 contains two highly conserved peptide sequences that negatively regulate Nrf2 activity by binding to β-transducin repeat-containing protein (β-TrCP). Neh6 domain also offers stability control of Nrf2 when Nrf2 is in the Nrf2-Keap1 complex. Nrf2 enters the nucleus and binds to the endogenous antioxidant reaction element (ARE) that can regulate downstream antioxidant enzymes, including NQO1, HO-1, etc. The Neh7 region inhibits the activation of Nrf2 by binding to the retinoid X receptor α (RXRα), a nuclear receptor that inhibits the Nrf2-ARE signaling pathway, to block the activation of Neh4 and Neh5 by activating factors (20). Nrf2 and its endogenous inhibitor, Keap1, function as a ubiquitous, evolutionarily conserved intracellular defense mechanism to counteract OS. Sequestered by cytoplasmic Keap1 and targeted to proteasomal degradation in basal conditions, in case of OS Nrf2 detaches from Keap1 and translocate to the nucleus, where it heterodimerizes with one of the small musculoaponeurotic fibrosarcoma proteins (sMaf). The heterodimers recognize the AREs, that are enhancer sequences present in the regulatory regions of Nrf2 target genes, essential for the recruitment of key factors for transcription.

Nrf2 function as a key transcription factor in the regulation of OS, involved in redox balance, drug metabolism and excretion, iron metabolism, amino acid metabolism, survival and proliferation, proteasome degradation and other physiological activities. It also participates in the regulation of cell cycle homeostasis, cytoprotection and innate immunity under cellular stress (21, 22). The main biological function of Nrf2 is to resist oxidative damage (23). The activation of Nrf2 activity is a treatment for diseases related to OS, and the antioxidant system involved is mainly composed of various endogenous antioxidants such as catalytic enzymes of glutathione (Glutathione peroxidase, GSH-Px), glutathione sulfhydryl transferases (GSTs), superoxide dismutase (SOD), catalase (CAT), peroxidase (PRDXs), and these antioxidants are almost all regulated by Nrf2 (24). The activation of Nrf2 mainly includes two pathways. The first classical activation pathway of Nrf2 is caused by OS (25). Specifically, Keap1 binds to Nrf2 in the cytoplasm and sequesters Nrf2 in an inactive state. Nrf2 is polyubiquitinated by the Keap1-Cul3 ubiquitin E3 ligase complex, resulting in rapid ubiquitin-proteasome-dependent degradation of Nrf2. However, during OS, the cysteine residues C273 and C288 of Keap1 are modified, resulting in a conformational change of Keap1, inactivating the E3 ligase function of Keap1, making the combination of Nrf2 and Keap1 unstable, and Nrf2 escapes proteasome degradation. Then excessive Nrf2 is released from the cytoplasm to the nucleus, forms a heterodimer with sMafs, and binds to ARE, ultimately inducing expression of cytoprotective genes including antioxidant genes and phase II enzymes (26, 27). Another non-classical activation pathway of Nrf2 is driven by autophagy dysfunction. Sequestosome-1(SQSTM1)/p62, a common receptor of selective autophagy, has the function to degrade ubiquitin substrates and participates in a variety of signal transduction pathways, including Keap1-Nrf2 pathway (28). Studies have shown that p62 competes with Nrf2 to bind Keap1 (29). This interaction enables p62 to chelate Keap1 into autophagosomes, thus preventing Keap1-mediated Nrf2 degradation and activating the Nrf2 pathway (30, 31).

Activation of Nrf2 can also be triggered through MAPK signaling molecules, such as p38 and JNK (32, 33). Besides, protein kinase C (PKC) was shown to phosphorylate the Neh2 structural domain of Nrf2 (34), leading to dissociation of Nrf2 from its repressor Keap1, thus promoting the transcriptional activity of Nrf2 (35). In addition, p21 belongs to the cyclin-dependent kinase inhibitor (CKI) family, which is directly positively regulated by the p53 gene and is involved in various physiological processes such as cell cycle arrest and OS (36). In the presence of OS, p21 gene expression is upregulated, which in turn promotes cell survival (37). Meanwhile, p21 upregulates Nrf2 protein levels by competitively binding Nrf2 with Keap1 to inhibit the Keap1-dependent ubiquitination degradation of Nrf2 (38).

## 3 Nrf2 in oncology

Studies have identified that Nrf2 is both a tumor suppressor and an oncogene. On one hand, Nrf2 can protect cells against endogenous and exogenous damage, and protect normal cells from OS by activating its downstream antioxidant genes against hazard chemical injury, so it is considered as a tumor suppressor (39). On the other hand, because OS is usually accompanied with the occurrence of cancer, tumor cells often show a variety of genetic changes and a high OS state, leading to excessive activation and persistent activation of Nrf2. Nrf2 helps cancer cells escape from ROS damage by expressing antioxidant target genes or promoting cancer cell survival and proliferation, and Nrf2 helps to prevent drug accumulation in cancer cells during radiotherapy and chemoradiotherapy resistance, thereby protecting cancer cells from apoptosis (40). Therefore, Nrf2 was described as a paradoxical protein with controversial suppressing and promoting roles in cancer cells. Generally, Nrf2 is lowly expressed in normal tissues and highly expressed in cancer, which guarantees Nrf2 as a marker that distinguishes normal tissue cells from cancer cells. Furthermore, clinical research studies have found that the prognostic ratio of the Nrf2 gene can be used as a robust poor predictor of various cancers. As a strong predictor, Nrf2 is also involved in deriving drug resistance in many human cancers (11). Although still controversial, it is undeniable that cancer cells with high Nrf2 activity and other carcinogenesis characteristics are closely related to drug resistance and tumor recurrence. The underlying mechanism is currently unclear but may involve oncoprotein biosynthesis, scavenging of ROS and toxic carcinogens (41). That is to say, Nrf2 can not only protect normal cells from harmful substances, but also promote the survival of tumor cells under cancer therapy (42). From the above-mentioned current state of Nrf2, we can boldly propose Nrf2 as a potential therapeutic target for different human cancers and provide new ideas for further development of new cancer therapies.

### 3.1 Nrf2 and digestive system cancers

#### 3.1.1 Nrf2 and colorectal cancer

Colorectal cancer (CRC) is a common malignancy that occurs in the digestive tract and amounts the third most common type of all gastrointestinal cancers (43). Shaoyao Decoction (SYD), a compound prescription of Chinese traditional medicine, was reported to have anti-colorectal cancer effect. Both *in vivo* and *in vitro* experiments demonstrated that SYD exerts antioxidant effect through activation of Nrf2 pathway and upregulation expression of Nrf2 downstream genes (γ-GCSc, NQO1). SYD is shown to have preventive effect against colitis-associated CRC (44). OS also plays a catalytic role in the progression of a range of gastrointestinal diseases, from chronic enteritis to CRC (45). Nrf2 is a protective ingredient against carcinogenesis and OS through upregulation of endogenous antioxidants and phase II antioxidant enzymes in CRC (46). Thus Nrf2 has become a new target for the prevention of CRC. In addition, Cyanidin Chloride (CyCl) is the active ingredient of mulberry, belonging to a type of anthocyanins (47). Studies shows that CyCl can inhibit the proliferation and induce apoptosis of CRC HCT116, HT29 and SW620 cells. CyCl can inhibit the NF-κB signaling pathway and induce the activation of the Nrf2 pathway in CRC cells stimulated by tumor factor TNF-α (48). Therefore, CyCl induces apoptosis by participating in NF-κB signaling in CRC cells, and Nrf2 may be a potential drug target to treat CRC by regulating the Nrf2/HO-1/NQO1 pathway (49).

#### 3.1.2 Nrf2 and gastric cancer

Gastric cancer (GC) is one of the most common types of cancer worldwide and has a very high mortality rate (50). Studies have shown that by utilizing *in vitro* and *in vivo* experiments, Brusatol, the inhibitor of Nrf2, after treatment with a gradient concentration, inhibits the expression of Nrf2/HO-1 axis down-regulating the expression of vascular endothelial growth factor (VEGF) and reduces its angiogenesis capacity (51). Protein expression of Nrf2, HO-1 and VEGF is reduced in a concentration-dependent manner. These studies demonstrate that Nrf2/HO-1 may be involved in the malignant process of GC formation by affecting angiogenesis (52). In addition, diallyl trisulfide (DATS) is a compound isolated from garlic with anti-tumor activity (53). DATS can inhibit the viability of GC BGC-823 cells and induce cell cycle arrest in G2/M phase in a dose-dependent manner. DATS decreased Akt phosphorylation in tumors, resulting in lower Nrf2 levels. DATS protects BGC-823 cells through the activation of p38 and JNK/MAPK and the weakening of Nrf2/Akt signaling pathway (54). It was found that Nestin was highly expressed in GC, and knockdown of Nestin reduced the viability of GC cell lines SGC-7901 and MKN-45, inhibited GC cell metastasis, induced apoptosis, decreased antioxidant enzyme production, and led to downregulation of Nrf2 expression (55). Besides, Nestin competed with Nrf2 to bind Keap1 and protected Nrf2 from Keap1-mediated degradation, thereby increasing the expression level of Nrf2, promoting cell viability and preventing apoptosis. The restoration of Nrf2 expression can counteract the inhibitory effects of Nestin knockdown on GC cell proliferation, migration, invasion and antioxidant enzyme production. In conclusion, the Nestin/Keap1/Nrf2 pathway can be utilized as a therapeutic target to inhibit the proliferation and metastasis of GC (56).

#### 3.1.3 Nrf2 and hepatocellular carcinoma

OS caused by alcohol drinking, hepatitis viral and eating habits is the main cause of liver cancer (57). Hepatocellular carcinoma (HCC) is the most common primary liver cancer and the fifth most common malignant cancer in the world. HCC tissue expresses more Nrf2 than para-carcinoma tissue. The targeted regulation of Nrf2 can be used to treat a variety of chronic diseases, including HCC (58).Raspberry extract is reported to significantly reduce ROS levels in H_2_O_2_-induced oxidatively damaged HCC HepG2 cells, increases GSH content and CAT activity, and activates the expression of the proteins Keap1, Nrf2, HO-1, NQO1, and γ-GCS through the Keap1/Nrf2 pathway (59). Meanwhile, epirubicin (EPI), a chemotherapeutic drug in clinical antineoplastic therapy (60), is detected the anti-cancer effects on HCC. In the initial reaction stage, EPI increases Nrf2 expression and intracellular ROS level, promotes Nrf2 up-regulation and nucleus translocation, thus aggregating tumor cell death. However, during the late stage, an excessive of ROS level can lead to the hyperactivation of Nrf2, through the overexpression of its downstream protective genes, and then promote the proliferation, invasion and metastasis of cancer cells, thus favorable for tumor cell survival. These pharmacological features of EPI indicate that when using EPI by single administration, its drug concentration and treatment time should be seriously concerned. To optimize EPI’s sophisticated efficacy, it is suggested to administrate EPI combined with Camptothecin (CPT), rather than administrating EPI alone. As an Nrf2 inhibitor, CPT can inhibit the expression of Nrf2 by down-regulating ROS, thereby inhibiting HCC cell proliferation and EMT process. Since CPT exhibits more confirmed tumor growth inhibitory effects than EPI, the co-administration of CPT and EPI can inhibit the ROS generation and Nrf2 over-expression initially induced by EPI, and can better inhibit the growth of HCC, compared with the single EPI administration. Therefore, CPT together with EPI play a synergetic tumor-inhibiting role. Conclusively, by down-regulating the expression of Nrf2, CPT combined with EPI can inhibit the proliferation, migration, invasion and angiogenesis of HCC cells (61) providing the evidence that Nrf2 can be used as a potential therapeutic target for the treatment of HCC.

#### 3.1.4 Nrf2 and pancreatic cancer

Pancreatic cancer (PC) is one of the deadliest and most aggressive malignant tumors, known as the “king of cancer” in the field of tumors (62). During pancreatic carcinogenesis, Nrf2 exerts tumor suppressive effects by binding to ARE and activating its downstream target genes (NQO1, SOD1, HO-1, ATF3, IL-17D, and SQSTM1/p62) that regulate cellular antioxidant/detoxification responses, immune surveillance and autophagy (63). PC’s chemical resistance which commonly leads to high recurrence rate is found closely related with the human HEAT repeat-containing protein 1 (HEATR1). HEATR1 competed with Keap1 for binding to SQSTM1/p62, resulted in up-regulation of Keap1, which then inhibited Nrf2 signaling in PC cells. Moreover, HEATR1 deficiency could promote PC proliferation and gemcitabine (GEM) resistance. In addition, HEATR1 deficiency significantly improved xenograft tumor growth *in vivo* by upregulating Nrf2 signaling (64). These results suggest a negative feedback correlation between HEATR1 and Nrf2/Keap1 pathway. Brucein D (BD), a naturally occurring quassinoid, is found able to enhance the chemosensitivity of GEM on PC through inhibition of the Nrf2 pathway, while the chemoresistance of GEM to PC cells can be significantly enhanced by silencing Nrf2. Mechanistic studies revealed that BD sensitized GEM in PC cells through the ubiquitin-proteasome-dependent degradation of Nrf2, and downregulated Nrf2 pathway. Silencing of Nrf2 plus BD treatment resulted in more potent inhibitory effects of GEM. In contrast, Nrf2 activation attenuated the chemosensitivity of GEM, indicating that the action of BD was Nrf2 dependent. In conclusion, BD was able to enhance the chemosensitivity of GEM in PC through inhibition of the Nrf2 signaling pathway (65).

#### 3.1.5 Nrf2 and esophageal squamous cell carcinoma

Esophageal squamous cell carcinoma (ESCC) is a deadly disease and one of the most aggressive cancers of the gastrointestinal tract (66). Nrf2 has been found to regulates the expression of enzymes involved in detoxification and anti-oxidative stress response signaling and protect ESCC cells from surviving in a highly oxidative environment. In clinical studies, Nrf2 was found to inhibit migration and invasion of ESCC cells in a hypoxic microenvironment. Additionally, NQO1, a downstream factor of Nrf2, has been found to enhance the antitumor effects of curcumin in ESCC xenograft tumors (67). NETO2 is an oncogene, and its overexpression induces EMT and cancer cell invasion and metastasis through activation of PI3K/Akt/NF-κB/Snail axis (68). The activation of ERK and Akt promotes Nrf2 expression, and overexpression of Nrf2 instead increases oncogenicity and chemoresistance (69). It was reported that downregulation of NETO2 reduced the proliferation and metastatic capacity of ESCC by regulating Nrf2 expression and PI3K/AKT/ERK pathway (70).

Based on previous researches, **Table 1** summarizes the mechanism of Nrf2 in digestive system tumors.

**Table 1:** Activities and mechanism of Nrf2 in digestive system cancers.

Tumor type	Cell lines	Signaling pathway	Effects	Reference
Colorectal cancer	HT-29 cells HCT116 cells, HT29 cells, SW620 cells	Nrf2/ARE NF-κB/Nrf2	Protective effect of ShaoYao decoction on colitis-associated colorectal cancer by inducing Nrf2 signaling pathway Cyanidin Chloride induces apoptosis by inhibiting NF-κB signaling through activation of Nrf2 in colorectal cancer cells	(44) (49)
Gastric cancer	BGC-823 cells AGS cells, MGC-803 cells	Nrf2/HO-1 Keap1/Nrf2	Nrf2/HO-1 axis regulates the angiogenesis of gastric cancer via targeting VEGF TCF7L1 indicates prognosis and promotes proliferation through activation of Keap1/Nrf2 in gastric cancer	(52) (71)
Hepatocellular carcinoma	Huh7 cells, H22 cells HepG2 cells	Keap1/Nrf2/ARE Keap1/Nrf2	Nrf2 down-regulation by camptothecin favors inhibiting invasion, metastasis and angiogenesis in hepatocellular carcinoma Protective effects of raspberry on the oxidative damage in HepG2 cells through Keap1/Nrf2-dependent signaling pathway	(59) (61)
Pancreatic cancer	Panc-1 cells, Canpan-2 cells Panc-1 cells, MiaPaCa-2 cells	Nrf2-ROS p62/Keap1/Nrf2	Brucein D augments the chemosensitivity of gemcitabine in pancreatic cancer via inhibiting the Nrf2 pathway HEATR1 deficiency promotes pancreatic cancer proliferation and gemcitabine resistance by up-regulating Nrf2 signaling	(64) (65)
Esophageal squamous cell carcinoma	TE-5TE-8 cells, TE-11R cells KYSE 30 cells, KYSE 150 cells, KYSE 450 cells, KYSE 410 cells, KYSE 510 cells, TE-1 cells	Nrf2/NQO1 Nrf2/SLC7A11	Combination treatment with highly bioavailable curcumin and NQO1 inhibitor exhibits potent antitumor effects on esophageal squamous cell carcinoma SLC7A11 regulated by Nrf2 modulates esophageal squamous cell carcinoma radiosensitivity by inhibiting ferroptosis	(67) (72)

### 3.2 Nrf2 and urinary system cancers

#### 3.2.1 Nrf2 and prostate cancer

Prostate cancer (PCa) is one of the most common urinary malignant tumors in men. PCa has a close relationship with Nrf2 (73). A study showed brain-type glycogen phosphorylase (PYGB) silencing suppressed the growth and promoted the apoptosis of PCa cells by affecting the NF-κB/Nrf2 signaling pathway. The results revealed that PYGB was upregulated in PCa tissues and PYGB silencing suppressed the cell viability of PC3 cells. PYGB silencing also increased the ROS content and affected NF-κB/Nrf2 signaling pathways. NF-κB has been shown to be associated with apoptosis, and both NF-κB and Nrf2 have anti-inflammatory and antioxidant effects. The dual interaction of PYGB silencing on NF-κB and Nrf2 jointly promotes apoptosis in PCa cells (74).

High glucose in the body can affect energy metabolism, cell proliferation, and increase ROS levels through glycolysis (75). It was reported that high glucose promoted LNCaP proliferation. Increased level of ROS in LNCaP cells under high glucose condition resulted in a decrease in downstream target proteins HO-1 and γ-GCS, all were transcripts of Nrf2 activation, thus triggering ROS-mediated subsequent inflammatory response, promoting the expression of pro-inflammatory factors IL-6 and IL-1b and reducing the content of anti-inflammatory factors IL-10, thereby increasing the expression level of apoptotic proteins, finally inducing the apoptosis of PCa cells (76, 77).

p62 is known to be a multidomain protein that can interact with Nrf2 to influence inflammation, OS, and the development of cancer (78). Studies reveal p62 promotes proliferation, apoptosis resistance and invasion of PCa cells via the Keap1/Nrf2/ARE pathway. p62 increased the levels and activities of Nrf2 by suppressing Keap1-mediated proteasomal degradation in PCa cells and tissues, and high levels of p62 promoted growth of PCa through accelerating the Keap1/Nrf2/ARE system and by activating Nrf2 pathway, p62 stimulates the transcription of Nrf2’s target genes, inhibits ROS in PCa cells, which promotes PCa cell proliferation, anti-apoptosis and invasion.

#### 3.2.2 Nrf2 and bladder cancer

Bladder cancer (BCa) is the second most common malignant tumor of the genitourinary system (79). In BCa, p62 serves as a selective autophagy adaptor, and also interacts with Keap1-Nrf2 pathway. Typically, p62 is overexpressed in BCa and promotes tumor growth through Keap1-Nrf2 signaling and protecting cancer cells from OS (80). Ailanthone (Aila), a natural compound with antitumor activity against various cancer cells (81), inhibits the proliferation, migration and invasion of BCa cells by reducing the expression of Nrf2, YAP and c-Myc (82). Another study reported that Berbamine induced cell cycle arrest in S-phase through activation of p21 and p27 protein expression and downregulation of CyclinD, CyclinA2 and CDK2 protein expression (83), and increased intracellular activity by down-regulating antioxidant genes such as Nrf2, HO-1, SOD2 and GPX-1 levels (84). In addition, MTX-211 is a potential antitumor agent that reduces GSH levels through the Keap1/Nrf2/GCLM signaling pathway (85), thereby effectively inhibits the proliferation of BCa cells. Therefore, regulating the metabolism of GSH through the Nrf2/GCLM signaling pathway may be an effective strategy for treating BCa or overcoming chemotherapy resistance (86, 87). Like above-mentioned PC and PCa, in BCa, p62 functions the same model with Nrf2 (88). p62 promotes the growth of BCa cells by activating the Keap1/Nrf2 pathway, while overexpression of NEDD4L inactivates the p62/Keap1/Nrf2 pathway. Summarily, NEDD4L has the ability to inhibit BCa cell growth and suppress p62/Keap1/Nrf2 pathway activity, which indirectly proves that NEDD4L/p62/Keap1/Nrf2 pathway may be an effective target for the treatment of BCa (89, 90).

#### 3.2.3 Nrf2 and renal cell carcinoma

Renal cell carcinoma (RCC), referred to as kidney cancer, belongs to one of the common malignancies of the urinary system (91). As one of the most important pathways to antioxidant stress, Nrf2-ARE signaling pathway influences the cell biology of RCC and the sensitivity of targeted therapy. Nrf2 was in a high expression state in RCC tumors and had long been proved to have carcinogenic effects (92). Therefore, it can be speculated that Nrf2 and downstream target genes may play a crucial role in the occurrence and development of RCC. Inhibiting Nrf2 expression significantly improved the RCC 786-0 cells to chemotherapy drugs resistance. By inhibiting the conduction of Nrf2-ARE signaling pathways to enhance the resistance of tumor cells to OS, the proliferation, migration and invasion of RCC cells were alleviated, and the resistance to chemotherapy drugs was reduced (93).

Above studies show that abnormal expression of Nrf2 is closely related to the occurrence of human urinary system tumors, including prostate, bladder and kidney cancer. The detailed evidences were summarized in **Table 2**.

**Table 2:** Activities and mechanism of Nrf2 in urinary system cancers.

Tumor type	Cell lines	Signaling pathway	Effects	Reference
Prostate cancer	LNCap cells, PC3 cells, DU145 cells, PrEC cells LNCaP cells DU145 cells RWPE-1 cells, PC3 cells	NF-κB/Nrf2 Nrf2/ARE Keap1/Nrf2/ARE Nrf2/NF-κB	Silencing of PYGB suppresses growth and promotes the apoptosis of prostate cancer cells via the NF-κB/Nrf2 signaling pathway High glucose promotes prostate cancer cells apoptosis via Nrf2/ARE signaling pathway p62 promotes proliferation,apoptosis-resistance and invasion of prostate cancer cells through the Keap1/Nrf2/ARE axis Nrf2 antioxidant pathway and apoptosis induction and inhibition of NF-κB-mediated inflammatory response in human prostate cancer PC3 cells by Brassica oleracea var. acephala: An *in vitro* study	(73) (74) (76) (94)
Bladder cancer	T24 cells, RT4 cells, 5637 cells, TCCSUP cells, 253J cells, SV-HUC‐1 cells, SW780 cells	p62/Keap1/Nrf2	p62 promotes bladder cancer cell growth by activating Keap1/Nrf2-dependent antioxidative response	(80)
Renal cell carcinoma	786-0 cells 786‐O cells, OS-RC-2 cells, Caki-1 cells, 769-P cells, A498 cells, ACHN cells, HK-2 cells	Nrf2/ARE Nrf2/TRIM24	Effect of the Nrf2-ARE signaling pathway on biological characteristics and sensitivity to sunitinib in renal cell carcinoma BMP8A promotes survival and drug resistance via Nrf2/TRIM24 signaling pathway in clear cell renal cell carcinoma	(93) (95)

### 3.3 Nrf2 and female reproductive system cancers

#### 3.3.1 Nrf2 and endometrial cancer

Endometrial cancer (EC) is the main gynecological malignancy, with most of cases occur in post-menopausal women and shows an increasing incidence in recent years (96). Aldo-Keto reductases family 1 (AKR1C1) is a key regulatory gene downstream of Nrf2. *In vitro* experiments confirmed that downregulation of Nrf2/AKR1C1 favors EC cells to be sensitive to progesterone. Overexpression of Nrf2 and AKR1C1 may be one of the main molecular mediators of progesterone resistance in patients with endometrial precancerous lesions and well-differentiated cancers. Thus, it’s rationale to reverse progesterone resistance in EC with the reduction of Nrf2/AKR1C1. Targeting the Nrf2/AKR1C1 pathway may represent a new therapeutic strategy for treatment of EC (97). Endometrial serous carcinoma (ESC) is a sub-type of EC, and SPEC-2 cells are a special EC cell line derived from ESC tumor tissue (98). Studies have shown that SPEC-2 cells express higher levels of Nrf2, and the expression of Nrf2 downstream genes including NQO1, HO-1, MRP2, GCLC and GCLM are increased in SPEC-2 cells. Furthermore, inhibition of Nrf2 expression made SPEC-2 cells more resistant to cisplatin and paclitaxel in a xenograft model. These results demonstrate the great promise of inhibiting Nrf2 to overcome chemotherapy resistance in EC (99).

#### 3.3.2 Nrf2 and ovarian cancer

The mortality rate of ovarian cancer (OC) ranks first among gynecological malignancies (100). Carboxymethylated pachyman (CMP), a traditional medicinal herb with antitumor activity (101), can induce ferroptosis in OC cells by suppressing Nrf2/HO-1/xCT/GPX4 (102). By targeting the Nrf2/Keap1/ARE pathway, CMP inhibits OC progression, proliferation and chemotherapy resistance. In addition, apatinib promotes apoptosis and autophagy in ROS-dependent OC cells by negatively regulating Nrf2 and p62 (103). Dihydrotanshinone I (DHT) is a compound extracted from the root of Danshen, which has many pharmacological activities such as anticancer (104), inhibits the viability of OC cells and induces OS through ubiquitination-mediated degradation of Nrf2, thereby exhibiting anti-OC effects (105).

#### 3.3.3 Nrf2 and cervical cancer

Cervical cancer (CC) is the most common gynecological malignancy in women worldwide (106). A study found that allicin, the main ingredient extracted from garlic, inhibits the proliferation and migration of CC cells by inhibiting the expression of Nrf2, thereby maintaining intracellular oxidative homeostasis (107). Detailed experimental data show that allicin inhibits the viability of CC SiHa cells in a time- and dose-dependent manner, and induces apoptosis. It was found Nrf2 overexpression can enhance SiHa cells migration and invasion, indicating Nrf2 has a carcinogenic effect in CC. In addition, treatment with allicin can significantly inhibits the expression of Nrf2 and the downstream antioxidant gene enzyme HO-1. Therefore, allicin mainly inhibits the malignant phenotype of CC cells by inhibiting the expression of Nrf2, which provides a clinical basis for the treatment of CC patients (108). In HeLa cells, metformin inhibits the expression of Nrf2 by attenuating Raf-ERK signaling through a Keap1-independent mechanism (109). Through inhibiting the expression of HO-1 downstream of Nrf2, metformin attenuates the proliferation of tumor cells and enhancing their sensitivity to anticancer drugs, indicating that the Raf/ERK/Nrf2 axis is a new molecular target for CC therapy (110).

By summarizing previous reports on Nrf2 and its clinical significance in female reproductive system, we found that some native small molecule compounds can also effectively inhibit the malignant phenotype of cancer cells by inhibiting the expression of Nrf2, and the detailed underlying mechanisms are shown in **Table 3** below (**Table 3**).

**Table 3:** Activities and mechanism of Nrf2 in female reproductive system cancers.

Tumor type	Cell lines	Signaling pathway	Effects	Reference
Endometrial cancer	RL95-2 cells SPEC-2 cells	Nrf2/AKR1C1 Keap1/Nrf2	Mechanism of progestin resistance in endometrial precancer/cancer through Nrf2-AKR1C1 pathway High levels of Nrf2 determine chemoresistance in type II endometrial cancer	(97) (99)
Ovarian cancer	HO8910PM cells, A2780 cells, SKOV3 cells, ES2 cells A2780 cells	Keap1/Nrf2 Nrf2/ABCF2	Dihydrotanshinone I inhibits ovarian tumor growth by activating oxidative stress through Keap1-mediated Nrf2 ubiquitination degradation ABCF2, an Nrf2 target gene, contributes to cisplatin resistance in ovarian cancer cells	(105) (111)
Cervical cancer	C33A cells, HeLa cells, SiHa cells HeLa cells	Nrf2/NQO1/GSH MBNL1/Nrf2	Role of Nrf2 cascade in determining the differential response of cervical cancer cells to anticancer drugs: an *in vitro* study MBNL1 regulates resistance of HeLa cells to cisplatin via Nrf2	(108) (112)

### 3.4 Nrf2 and other cancers

#### 3.4.1 Nrf2 and breast cancer

Breast cancer (BC) is the most common malignancy among women all around the world (113). Due to the high heterogeneity, BC’s pathogenesis has not been adequately established, and its treatment remains a serious challenge in healthcare systems (114). The overexpression of Nrf2 enhances proliferation and migration of BC cells such as MCF‐7 and MDA‐MB‐231. Mechanically, Nrf2 promotes the expression of G6PD and HIF‐1α. G6PD could be an anticancer target that is associated with prognosis in a variety of cancers (115). Overexpression of Nrf2 up‐regulated the expression of Notch1 via G6PD/HIF‐1α pathway. G6PD is a rate-limiting enzyme in the pentose phosphate pathway (PPP), and G6PD overexpression is related to cancer prognosis and tumor metastasis (116). Notch signaling pathway affected the proliferation of BC by affecting its downstream gene HES‐1, and regulated the migration of BC cells by affecting the expression of EMT pathway. All these results shows that Nrf2 is a potential molecular target for the treatment of BC (117). As a potential tumor suppressor (118), miR-101 can increase its expression level and reduce the protein expression level of Nrf2 in the nucleus. Then the lowed Nrf2 in turn leads to the decreased of the activity of the antioxidant signaling pathway Nrf2/ARE, so that Nrf2’s downstream antioxidant protein expression is declined, and the sensitivity to OS is also impaired, resulting in an oxidation and antioxidant imbalance in cancer cells, and inducing the apoptosis of BC cells. Finally, miR-101 achieves the inhibitory effect to BC cells proliferation through its suppression on Nrf2 signaling pathway (119).

Levistilide A (LA), an active compound extracted from Chuanxiong Rhizoma, has been shown that it has anti-cancer effects (120). Specifically, LA can induce mitophagy in MDA-MB-231 and MCF-7 cells in a dose-dependent manner, mainly by promoting the overexpression of Nrf2/HO-1, thereby causing excessive accumulation of ROS in BC cells, and eventually lead to cell ferroptosis (121).

HIF-1α glycolytic pathway plays a central role in a study of Nrf2 promoting BC cell growth by enhancing glycolysis (122). In MCF-7 and MBA-DA-231cells, the expression of Nrf2 was positively correlated with the expression of glycolytic genes, and the up-regulation of Nrf2-mediated glycolytic enzymes was dependent on the activation of AKT and the inhibition of AMPK, thereby promoting proliferation of cancer cells. Comparatively, mutations in the tumor suppressor gene BRCA1 are known to predispose BC (123, 124), while BRCA1-deficient cells accumulate ROS due to a defect in Nrf2, and reactivation of Nrf2 can rescue cell survival (125). It is shown that estrogen (E2) acts through the PI3K-AKT pathway to regulate the activation of Nrf2 in BRCA1-deficient cells (126, 127), thereby inducing the production of antioxidant genes including GCLM and HO-1, protecting cells from ROS-induced death and enabling the survival of BC cells (128). In conclusion, Nrf2 promotes BC progression, implicating that Nrf2 is a potential molecular target for BC treatment.

#### 3.4.2 Nrf2 and lung cancer

Lung cancer is the most common cause of cancer-related death worldwide (129). Cyclin-dependent kinase 20 (CDK20) is a cell cycle regulatory factor (130). In lung cancer cells, CDK20 can compete with Nrf2 to bind to Keap1, enhance the transcriptional activity of Nrf2 and reduce the level of ROS, thereby promoting the continuous proliferation of cancer cells (131). When the PI3K/AKT/mTOR pathway is continuously activated, the activity of Nrf2 is further enhanced, which induces metabolic reprogramming of cells and promotes the abnormal proliferation of A549 cells. Therefore, the interaction between Nrf2 and PI3K/AKT/mTOR signaling pathway plays a significant role in promoting the malignant progression of lung cancer (132), and the elevated Nrf2 expression promotes lung cancer progression and enhances the ability of tumor cells to evade apoptosis (133).

Noteworthily, during the process of lung cancer development, Nrf2 plays an inhibitory role in the beginning stage of cancer and a pro-cancer role in the development stage. Nrf2 deficient specimens exhibit higher tumor incidence in a mouse model of lung cancer. In turn, after lung carcinogenesis, Nrf2 promotes the transformation of benign adenomas into malignant adenomas and accelerates lung cancer progression through the Kras/Nrf2/GPX2 and MRP4 pathways (134, 135). These results suggest that Nrf2 prevents cancer in the early stages, while accelerates cancer progression in the late stages of lung carcinogenesis (136).

#### 3.4.3 Nrf2 and glioblastoma

Glioblastoma (GBM) is the most common and aggressive malignancy of the central nervous system (CNS) (137, 138). An investigation explores the action of Nrf2 in GBM U251 cells and reveals the collaboration between Nrf2 and matrix metalloproteinase 9 (MMP9), which is a positive indicator for tumor cell migration and invasion (139). Detailly, upregulation of Nrf2 led to an increase in MMP9 expression and activity whereas downregulation of Nrf2 led to a decrease in MMP9 expression and activity, and Nrf2 significantly provoked GBM cell migration and invasion (140). In addition, apatinib, an anti-angiogenic drug (141), can block the cell cycle in G0/G1 phase and inhibit the growth of glioma cells U251 and U87 by inducing ferroptosis. Nrf2-related anti-OS is closely related to ferroptosis inhibition (142), and apatinib can promote ferroptosis in GBM cells by regulating Keap1/Nrf2/VEGFR2 signaling pathway. Interestingly, excessive Nrf2 could reverse the inhibition of apatinib-induced GBM cell proliferation and the induction of ferroptosis (143). Like in lung cancer during different progression stage, this interesting phenomenon of Nrf2 in GBM exemplifies that Nrf2 function dual-effects both “good” or “bad” in cancer, and both effects display equal importance.

#### 3.4.4 Nrf2 and osteosarcoma

Osteosarcoma (OS), a mesenchymal malignancy, is the most common primary tumor of bone, and is the third most common type of cancer among children and adolescentsbetween the ages of 12 and 18 (144). Members of the tripartite motif (TRIM) family proteins are known to act as tumor suppressor genes or oncogenes to influence the proliferation and differentiation of tumor cells and apoptosis (145). TRIM22 is a member of the TRIM family proteins (146). The malignant phenotype of tumors can be suppressed by disrupting and promoting the degradation of Nrf2. TRIM22 inhibited OS progression through Nrf2-mediated intracellular ROS imbalance. ROS production was significantly promoted when overexpressing TRIM22, thus activating AMPK/mTOR signaling. Meanwhile, TRIM22 inhibits OS progression by promoting proteasomal degradation of Nrf2, thereby activating signaling that leads to autophagic cell death in OS. Therefore, targeting TRIM22/Nrf2 could be a promising therapeutic strategy for treating OS (147).

#### 3.4.5 Nrf2 and leukemia

Leukemia is a malignant tumor of the hematopoietic system (148). It is known that cell cycle is closely related to proliferation (149), among which cdc2/cyclin B is a complex that controls G2/M cell cycle transition (150). PBK/TOPK is a substrate of cdc2/cyclin B that can promote mitosis (151), and PBK/TOPK protein is upregulated in a variety of hematological malignancies, which is important for the proliferation and malignant transformation of hematological tumors (152). Mitochondrial dysfunction and ROS production are involved in PBK/TOPK-induced G2/M cell cycle arrest, apoptosis, and inhibition of promyelocyte proliferation. The down-regulation of Nrf2 induces the decrease of cdc2 and cyclin B protein expression through the down-regulation of PBK/TOPK, which leads to cell cycle arrest and apoptosis of acute myeloid leukemia (AML) cells (153). In addition, HO-1 and Nrf2 protect against the deleterious effects of inflammation and OS, but may also contribute to protect oncogenic AML cells from TNF-mediated cell death by activating Nrf2 to induce HO-1 to inhibit TNF induced AML cell death (154).

We summarize the signaling pathways and mechanisms involved in the role of Nrf2 as an anti-cancer therapeutic target in BC, lung cancer, GBM, OS and leukemia, as shown in **Table 4**.

**Table 4:** Activities and mechanism of Nrf2 in other cancers.

Tumor type	Cell lines	Signaling pathway	Effects	Reference
Breast cancer	MDA-MB-231 cells, MCF-7 cells MDA-MB-231 cells, MCF-7 cells MCF-10A cells, MDA-MB-231 cells, MCF-7 cells MCF-7 cells	Nrf2/HO-1 Nrf2/G6PD/HIF-1α Nrf2/HIF-1α Nrf2/ARE	Levistilide A induces ferroptosis by activating the Nrf2/HO-1 signaling pathway in breast cancer cells Nrf2 promotes breast cancer cell migration via up-regulation of G6PD/HIF-1α/Notch1 axis Nrf2 facilitates breast cancer cell growth via HIF-1α-mediated metabolic reprogramming Effect of miR-101 on proliferation and oxidative stress-induced apoptosis of breast cancer cells via Nrf2 signaling pathway	(117) (119) (121) (123)
Lung cancer	SQ-19 cells, H2126 cells, H1437 cells, H1395 cells, A549 cells NCI-H1299 cells, NCI-H460 cells	Keap1/Nrf2 Nrf2/ARE	Loss of Keap1 function activates Nrf2 and provides advantages for lung cancer cell growth Nrf2/ARE pathway activation is involved in negatively regulating heat-induced apoptosis in non-small cell lung cancer cells	(155) (156)
Glioblastoma	T98G cells U251 cells U87MG cells, U251 cells	Nrf2/ABCC1/MRP1 Nrf2/MMP9 AKT/Nrf2/HO-1	High levels of Nrf2 sensitize temozolomide-resistant glioblastoma cells to ferroptosis via ABCC1/MRP1 upregulation The role of Nrf2 in migration and invasion of human glioma cell U251 The E3 ubiquitin ligase NEDD4-1 mediates temozolomide-resistant glioblastoma through PTEN attenuation and redox imbalance in Nrf2-HO-1axis	(140) (157) (158)
Osteosarcoma	HOS cells, Saos-2 cells, 143B cells, U2OS cells, MG63 cells, hFOB 1.19 cells MG63 cells	Nrf2/TRIM22 AMPK/Nrf2	TRIM22 inhibits osteosarcoma progression through destabilizing Nrf2 and thus activation of ROS/AMPK/mTOR/autophagy signaling Tanshinone IIA inhibits osteosarcoma growth through modulation of AMPK-Nrf2 signaling pathway	(147) (159)
Leukemia	HL-60 cells, THP-1 cells THP-1 cells, U937 cells, MV4-11 cells	Nrf2/STAT3 Nrf2/RFC4	LncRNA GAS5 induces cell apoptosis in acute myeloid leukemia by targeting Nrf2 Nrf2 overexpression increases the resistance of acute myeloid leukemia to cytarabine by inhibiting replication factor C4	(150) (160)

## 4 Nrf2-mediated double-edged mechanism

### 4.1 Activation of Nrf2 as anti-carcinogenic role

As mentioned earlier, Nrf2 is an important target for chemopreventive strategies in common human solid tumors because of its ability to promote the expression of detoxifying enzymes and cytoprotective genes. Therefore, inducers and inhibitors of Nrf2 are considered important cancer chemopreventive agents. The increase in ROS leads to NF-κB activation, while Nrf2-mediated stimulation of antioxidant pathways leads to a decrease in ROS, so the activation of Nrf2 leads to the production of antioxidant enzymes HO-1, UGT, NQO1, and GST, which leads to NF-κB inhibition. Therefore targeting Nrf2 and NF-κB pathways to reduce OS and inflammation is a very useful chemopreventive tool to prevent inflammation-related cancers.

Before tumorigenesis, Nrf2 activation exerts a chemical defense thereby inhibiting tumorigenesis (161), regulating the expression of downstream phase II detoxification enzymes and antioxidants, mainly mediated by the Nrf2-Keap1-ARE pathway (162, 163). These enzymes and antioxidants protect the body and cells from ROS and toxic substances such as carcinogens. At this circumstance, Nrf2 attenuates the expression of oncogenes, inhibits cell proliferation and angiogenesis, etc., thus providing a protective effect (164, 165). The tumor suppressive effects of Nrf2 have been studied mainly through many *in vivo* experiments. Nrf2 plays an important role in chemoprevention, and studies have compared the susceptibility of Nrf2 knockout mice with wild-type mice to chemically induced carcinogenic effects in which Nrf2 knockout mice are more susceptible to cancer when exposed to chemical carcinogens, such as bladder, skin and stomach cancers (166). Nrf2-deficient mice are more susceptible to chemical carcinogens, mainly associated with low basal expression of phase II detoxification enzymes, such as low total GST and NQO1 enzyme activity in the stomach (167).

### 4.2 Hyperactivation of Nrf2 as pro-carcinogenic role

In the opposite situation other than upregulating cytoprotective genes in normal cells, aberrant Nrf2 activation leads to increased antioxidant capacity in cancer cells. The overactivation of Nrf2 can help cancer cells escape OS by expressing antioxidant target genes or directly promoting cell survival and proliferation. In addition, Nrf2 plays an important role in radiotherapy resistance, preventing the accumulation of drugs in cancer cells, thus protecting cells from apoptosis (168). Among them, Nrf2-mediated pro-carcinogenic mechanism mainly promotes the growth and proliferation of tumor cells by increasing the expression of metabolic enzymes such as G6PD, TKT, and PGD, which promote glucose and nucleic acid metabolism, thus promoting the growth and proliferation of tumor cells (169). Furthermore, Nrf2 can interact with apoptosis-related genes p53 and Bcl-2 to promote tumorigenesis and sustained deterioration (170), and prevent free iron accumulation by controlling the expression of MT1, ferritin, and iron transport proteins, thus preventing iron accumulation (171, 172).

The role of Nrf2 in drug resistance is based on Nrf2-induced genes such as its downstream detoxifying and antioxidant enzymes HO-1 and NQO1. These Nrf2-induced genes allowed cells protected from the cytotoxic effects of anticancer therapies and resist apoptosis, which in turn triggers drug resistance in tumor cells (173, 174). Chrysin (5,7-dihydroxyflavone) is a potent Nrf2 inhibitor (175). By down-regulating PI3K-Akt/Nrf2 and ERK/Nrf2 signaling pathways, Chrysin significantly reduces the expression of Nrf2 at the mRNA and protein levels in ADM BEL-7402 cells through suppressing the expression of Nrf2 downstream genes HO-1, AKR1B10 and MRP5 (176, 177), and inhibit Nrf2-dependent chemotherapy resistance (178). Nrf2 enhancement of CSCs may be one of the reasons why tumors develop drug resistance (179). Nrf2 has been reported to protect normal cells from telomerase replication or oncogene-induced senescence through multiple mechanisms that prolong lifespan and reduce the expression of ROS, nuclear alterations, DNA damage, and senescence-associated β-galactosidase, thereby stimulating tumor progression (180). Besides, Nrf2 promotes cancer invasion and metastasis by regulating the expression of HIF-1α, VEGF, PDGF, E-cadherin, MMP-2, in tumor cells (181–184). Nrf2 was upregulated in gallbladder carcinoma (GC) tissues and acted as an independent prognostic factor (185). Propofol, one of the mostly used intravenous anesthetics, induces the proliferation and invasion of GC cells by activating Nrf2 (186). In addition, activation of Keap1-Nrf2 promotes cell migration, invasion and OS resistance in metastatic cancer cells (187).

Collectively, Nrf2 exerts pro-carcinogenic role mainly through promoting tumor cell growth and proliferation, inhibiting tumor cell apoptosis and ferroptosis, enhancing the drug resistance of tumor cells, stimulating tumor cells self-renewal, exerting unlimited replication potential, maintaining persistent angiogenesis, and involved in the invasion and metastasis of many cancers.

## 5 Agents targeting Nrf2’s therapeutic role in human cancers

Whether the activation state of the Nrf2 signaling pathway is normal or excessive will modulatory determine its dual role of chemopreventing and promoting cancer development. On one hand Nrf2 has a chemopreventive effect on carcinogenesis in normal organisms. On the other hand Nrf2 aberrant expression and persistent activation can promote cancer development. Therefore, Nrf2 activators that increase Nrf2 activity are used for disease prevention, while Nrf2 inhibitors are used to improve the efficacy of chemotherapeutic agents, so the search for compounds or drugs that activate the Nrf2 signaling pathway in the normal body and inhibit it in tumors is also the research focus.

### 5.1 Nrf2 activators

Based on the dual effects of Nrf2 promoting the survival of normal cells and inhibiting chemocarcinogenesis, it is rationale to propose Nrf2 activators as cytoprotective and cancer-preventing therapeutics (188). Nrf2 pathway is crucial for cellular defense against endogenous and exogenous OS and electrophilic stress (189). Actually, some Nrf2 activators’ anti-cancer effects have been partially elucidated. Nrf2 activators can accelerate the detoxification of carcinogens from the environment and protect the body from chemical carcinogenesis, and are used to protect normal cells from carcinogens. Among them, the only FDA-approved Nrf2 inducer is dimethyl fumarate (DMF), which has been used to treat multiple sclerosis and plays a protective role in various neurological diseases (190). The protective effect of DMF has been shown to be exerted by activating Nrf2/HO-1 signaling and enhancing GSH and TAC levels (191). Studies showed that the anticancer mechanism of DMF is paradoxically related to the decrease in the nuclear translocation of Nrf2 and indicated the potential therapeutic role of DMF in cancers (192). However, long-term use of DMF can also cause some gastrointestinal side effects (193), so the effectiveness of using Nrf2 activators in the treatment of malignant tumor needs to be further verified to avoid the harmful effects of Nrf2.

There are also some natural compounds originated from plant extracts showing Nrf2 activation effects. Sulphoraphane (SFN), mainly derived from *broccoli* and other cruciferous vegetables (194), can reactivate cellular resistance by inducing Nrf2/ARE/Prdx6 activity during aging and OS (195). In addition, SFN activates AMPK signaling by inducing excessive ROS production, promotes Nrf2 translocation, and leads to inhibition of PC cell viability (196). As a Nrf2 activator, SFN’s anticancer activity has been validated in cancers such as lung, prostate, breast and colorectal cancer (197).

Besides, curcumin is another non-toxic natural small molecular compound extracted from *turmeric* with multiple biological activities such as anti-oxidation, anti-cancer and anti-inflammation (198), and its pharmacological mechanism has been proven to affect the activity of Nrf2 signaling pathway. Curcumin down-regulates the expression of Fen1 in an Nrf2-dependent manner (199), thereby inhibiting the proliferation of BC cells (200). Specifically, curcumin includes Keap1 inhibiting, up-regulates the expression of Nrf2 and its target proteins, and enhances Nrf2 nuclear translocation (201). The detailed signaling pathway after curcumin treatment involves PI3K/Akt-1/mTOR, Ras/Raf/MEK/ERK, GSK-3β and p53 pathway, and these involved survival pathways will be finally transduced by NF-κB, Akt, Nrf2/ARE pathway (202). However, the relationship between curcumin and Nrf2 has not been thoroughly and systematically studied in human tumors, and further clinical research is still needed.

### 5.2 Nrf2 inhibitors

Since nuclear Nrf2 overexpression and the whereafter aberrant activation of Nrf2 have been observed in several human cancers, inhibition of Nrf2 has been considered as an anticancer strategy, and several Nrf2 inhibitors with different mechanisms of action have been discovered (203, 204).

Several natural small molecular compounds have a significant inhibitory effect on Nrf2. CyCl, CMP, Brusatol and allicin can effectively inhibit the malignant phenotype of tumor cells mainly by inhibiting the expression of Nrf2 and its downstream regulators (205). Among them, Brusatol acts as an inhibitor of the Nrf2 pathway, selectively reducing the protein level of Nrf2 by enhancing the ubiquitination and degradation of Nrf2. Brusatol inhibits Nrf2 protein levels in various cell types including A549, HeLa, MDA-MB-231, Ishikawa and SPEC-2, reduces Nrf2 downstream protein expression, and inhibits Nrf2-dependent protective responses (206). These natural Nrf2 inhibitors can therefore be used to enhance the efficacy of various chemotherapy drugs to treat many types of cancer. Discouragingly, in clinical studies, Brusatol was found to cause adverse reactions such as hypotension, nausea and vomiting (207). This can be explained that Nrf2 is widely expressed in normal cells, and using Nrf2 as a target for tumor therapy should have some side effects. Like tyrosine kinase inhibitors (TKIs) for epidermal growth factor receptor (EGFR), a classical and effective therapeutic target for NSCLC therapy, although the drugs erlotinib and gefitinib are very effective in treating NSCLC, they indeed cause side effects such as rash and diarrhea (208). This fact suggests using Nrf2 inhibitors for clinical application when treating Nrf2 as therapeutic target, the systemic toxicity and side effects should be carefully examined.

Luteolin, a flavonoid compound, is also a potent Nrf2 inhibitor, which mainly inhibits Nrf2 activity through the AKT/PI3K pathway. Luteolin induced the degradation of Nrf2 mRNA in A549 cells (209), resulting in the downregulation of Nrf2 target genes and increased sensitivity of A549 NSCLC cells to chemotherapy drugs (210).

In Nrf2-activated tumors, inhibition of Nrf2 has emerged as an attractive therapeutic strategy to combat this acquired resistance. ML385 is an inhibitor of the Nrf2-Keap1 pathway. It mainly interacts with the transcriptional activity of ARE and prevents the combination of the two, thereby inhibiting Nrf2 and overcoming the drug resistance of NSCLC cells due to Keap1 deficiency to carboplatin and other chemotherapy drugs (211).

In addition, Halofuginone (HF) is a promising Nrf2 inhibitor (212). HF significantly reduced the viability of cancer cells, caused severe hematopoietic and immune cell suppression in a dose-dependent manner (213). By taking a nanomedicine approach and encapsulating HF into polymer micelles (HF micelles; HFm), the systemic toxicity exhibited by free HF can be mitigated while maintaining antitumor properties. Therefore, HFm can effectively eradicate Nrf2-activated lung adenocarcinoma, and is a potent inhibitor without any obvious toxicity, which may play an important role in the clinical setting in the future (214).

Overactivation of Nrf2 promotes the growth and proliferation of cancer cells, blocks apoptosis, enhances the self-renewal ability of CSCs, and most importantly enhances the chemoresistance and radioresistance of cancer cells. So blocking Nrf2 activity in tumor cells is an important way to prevent cancer. However, an ideal inhibitor for clinical application not only requires effective efficiency and specificity, but also requires low toxicity, good biological activity and pharmacokinetics, and needs to be explored further.

## 6 Conclusion and prospective

The formation of cancer is closely related to OS. The overall relationship is that continuous OS leads to chronic inflammation, and inflammation finally promotes cancer (215). Chronic inflammation and OS are interrelated pathological processes that can lead to the initiation and progression of cancer. Nrf2, as a transcription factor that regulates the redox state of cells, and a major regulator of cellular ROS and chemical detoxification, not only participates in the chemoprevention of normal cells, but also promotes the growth of cancer cells. Keap1/Nrf2/ARE is an important signaling pathway to react OS, regulates the transcription of many antioxidants, activates a series of defense systems, and blocks or reverses cancer occurrence by inhibiting the activation of carcinogens or inducing the detoxification of phase II metabolic enzymes. When the intracellular ROS increases, the Nrf2 system is activated, and the cells express more antioxidant enzymes that synthesize antioxidants. The enhanced effect of the antioxidant system reduces the production of ROS, thereby achieving redox balance.

Activation of Nrf2 has a dual role in cancer. First, Nrf2 can regulate the transcription of many antioxidants and activate a series of defense systems through the Keap1/Nrf2/ARE antioxidant system, maintaining cellular redox homeostasis. Nrf2’s anti-inflammatory and anti-cancer activities avoid the body from causing damage, thus benefit the survival of normal cells. For this aspect, the activation of Nrf2 is very important in cancer prevention. Second, overactivation of Nrf2 also protects cancer cells and promotes their growth. Excessive activation of the Nrf2 signaling pathway plays a tumor-promoting role mainly by maintaining proliferation signals, infinite replication, continuous angiogenesis, resistance to apoptosis and ferroptosis, escape from immune destruction, and promotion of invasion and migration (216).

The fact that Nrf2 pathway plays a key role in inflammation and OS-mediated carcinogenesis assures Nrf2 pathway as a potential target for mediating tumor progression and survival (217). By targeting and inhibiting antioxidant pathways such as Nrf2 or Nrf2-ARE, an increase amount of ROS in the tumor microenvironment will be induced, then some key tumor signal transduction pathways will be inhibited. As a result, programmed cell death will occur, and tumor cells will be killed. Nrf2 is currently targeted through two strategies: Nrf2 activators can be used to prevent chemocarcinogenesis while Nrf2 inhibitors can be used for cancer therapy. Theoretically, no matter Nrf2 activators, or Nrf2 inhibitors, both these two strategies will eventually achieve the cancer therapeutic destination. Practically, according to previously published research reports, the development of Nrf2 pathway inhibitors may provide better strategies for cancer prevention and treatment. However, how to properly regulate Nrf2 with refraining its overactivation still needs follow-up studies. Overall, Nrf2 activation is not clearly defined as “good” or “bad”. Therefore, it is necessary to clarify whether the activation and inhibition of the Nrf2 pathway will have beneficial or adverse effects on the organism, as well as the complex relationship between them, so as to use different targeting strategies for different cancers (58).

In conclusion, Nrf2 is traditionally a tumor suppressor, and its cytoprotective function is thought to be the primary cellular defense mechanism against exogenous and endogenous damage, including exogenous stimuli and OS. However, Nrf2 hyperactivation creates an environment conducive to the survival of tumor cells, protecting tumor cells from OS, radiotherapy and chemotherapy. In addition, Nrf2 is mostly highly expressed in different types of tumors, participates in the regulation of redox homeostasis, and is associated with the tumor progression, aggressiveness, treatment resistance, poor prognosis, and expression of various oncogenes etc. In these cancers, for example, in lung carcinogenesis, Nrf2 plays different roles at different stages, making Nrf2 involved in the dual role in cancer. Therefore, Nrf2 acts differently at different stages of the same cancer development cycle, with Nrf2 having an inhibitory effect during the cancer initiation stage and a promoting effect at the late stage. On one hand, many drugs (or genetic alterations) that enhance Nrf2 activity can inhibit cancer development; on the other hand, genetic deletion of Nrf2 can promote cancer development, while Nrf2 has an oncogenic effect from the developmental stage.

**Figure 2:**
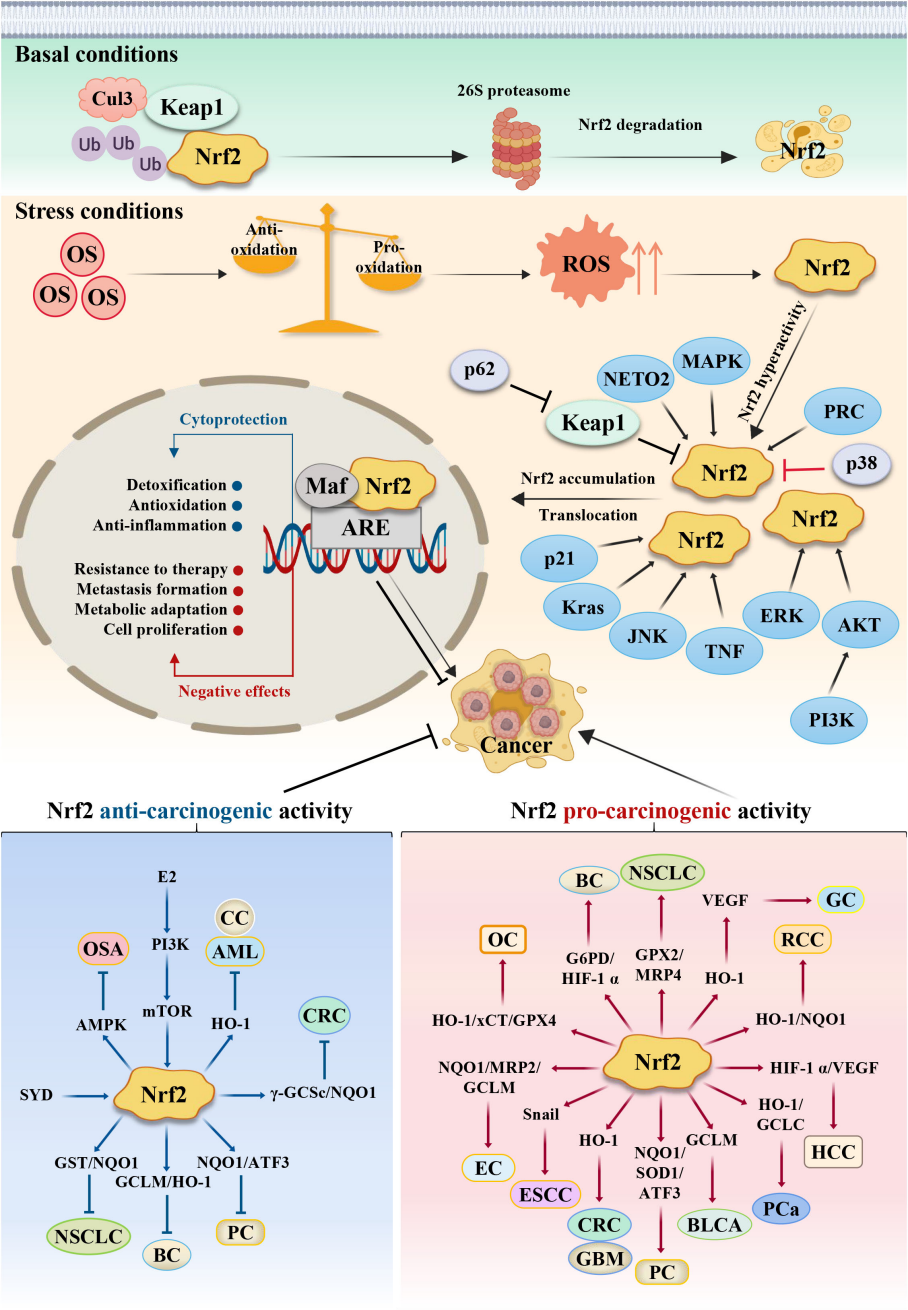
Schematic model of the action mechanism of Nrf2. Persistent OS leads to the development of cancer. Under basal conditions, Nrf2 protein is maintained at low levels in the cytoplasm via the E3 ubiquitin ligase Keap1, which regulates the ubiquitination degradation of Nrf2 through the proteasome pathway. Meanwhile, antioxidants outweigh pro-oxidants; but under stress conditions, pro-oxidants outweigh antioxidants, which results in ROS accumulation and Nrf2 activation. Under this stress conditions, the cysteine residues in Keap1 are oxidized, leading to Keap1’s dissociation from Nrf2, resulting in stabilization and translocation of the Nrf2 protein from cytoplasm to nucleus for binding to the antioxidant response element (ARE), primarily involving the expression of some cytoprotective and negative effective genes. Nrf2’s dual role in different stage confers different reaction. Some cancer cells can escape endogenous tumor suppression through activation of Nrf2, thus Nrf2 exerts the pro-carcinogenic activity, while some other cancer cells can’t overcome the tissue protection effects to normal healthy cells, thus normal healthy cells will proliferate greatly, which competitively suppresses cancer cells growth. At this situation, Nrf2 protects normal healthy cells and exerts the anti-carcinogenic activity. CRC, Colorectal cancer; GC, Gastric cancer; HCC, hepatocellular carcinoma; PC, Pancreatic cancer; ESCC, Esophageal squamous cell carcinoma; PCa, Prostate cancer; BLCA, Bladder cancer; RCC, Renal cell carcinoma; EC, Endometrial cancer; OC, ovarian cancer; CC, Cervical cancer; BC, Breast cancer; NSCLC, Lung cancer; GBM, Glioblastoma; OS, Osteosarcoma; AML, Leukemia. SYD, Shaoyao Decoction; E2, estrogen.

Based on the literatures about Nrf2 reported by previous researchers, in this review we summarize the different types and mechanisms of common tumors in which Nrf2 is involved. This review mainly focuses on the Keap1/Nrf2/ARE signaling pathway and its downstream genes, as well as the expression of related signaling pathways involving AKT/AMPK, PI3K/AKT/mTOR and NF-ĸB, and so on, thereby affecting the malignant biological behavior of tumor cells. All these unique role and mechanism are schematically represented in **Figure 2** to show the dual effects of Nrf2 in cancer progression and therapeutics. Summarily, our research depicts some of the known mechanisms by which Nrf2 can exert its double-edged pro-carcinogenic and anti-carcinogenic functions, and describes the current findings of Nrf2 activators and inhibitors, providing a clear rationale for the consideration of Nrf2 as a powerful putative therapeutic target in cancer treatment. At the same time, the regulation of Nrf2 expression is related to cancer chemotherapy, molecularly targeted therapy and immunotherapy. An in-depth understanding of the relationship between Nrf2 and tumors will further provide a theoretical basis for clinical use, as well as new strategies for cancer therapy.

## Author contributions

LL, QW, and FL drafted the manuscript. All authors contributed to the article and approved the submitted version.
